# Cardiomyocyte growth and sarcomerogenesis at the intercalated disc

**DOI:** 10.1007/s00018-013-1374-5

**Published:** 2013-05-26

**Authors:** Amanda J. Wilson, Roman Schoenauer, Elisabeth Ehler, Irina Agarkova, Pauline M. Bennett

**Affiliations:** 1 Randall Division of Cell and Molecular Biophysics, King’s College London, New Hunt’s House, Guy’s Campus, London, SE1 1UL, UK; 2 Institute of Cell Biology, ETH Zurich, Zurich, Switzerland; 3 Present Address: Department of Bioengineering, Imperial College London, South Kensington Campus, London, UK; 4 Present Address: Institute of Anatomy, University of Bern, Switzerland; 5 Present Address: Institute for Biomechanics, ETH Zurich, Zurich, Switzerland

**Keywords:** Heart structure, Dilated cardiomyopathy, Adherens junction, Electron microscopy, Transitional junction

## Abstract

Cardiomyocytes grow during heart maturation or disease-related cardiac remodeling. We present evidence that the intercalated disc (ID) is integral to both longitudinal and lateral growth: increases in width are accommodated by lateral extension of the plicate tread regions and increases in length by sarcomere insertion within the ID. At the margin between myofibril and the folded membrane of the ID lies a transitional junction through which the thin filaments from the last sarcomere run to the ID membrane and it has been suggested that this junction acts as a proto Z-disc for sarcomere addition. In support of this hypothesis, we have investigated the ultrastructure of the ID in mouse hearts from control and dilated cardiomyopathy (DCM) models, the MLP-null and a cardiac-specific β-catenin mutant, cΔex3, as well as in human left ventricle from normal and DCM samples. We find that the ID amplitude can vary tenfold from 0.2 μm up to a maximum of ~2 μm allowing gradual expansion during heart growth. At the greatest amplitude, equivalent to a sarcomere length, A-bands and thick filaments are found within the ID membrane loops together with a Z-disc, which develops at the transitional junction position. Here, also, the tops of the membrane folds, which are rich in αII spectrin, become enlarged and associated with junctional sarcoplasmic reticulum. Systematically larger ID amplitudes are found in DCM samples. Other morphological differences between mouse DCM and normal hearts suggest that sarcomere inclusion is compromised in the diseased hearts.

## Introduction

**Fig. 1:**
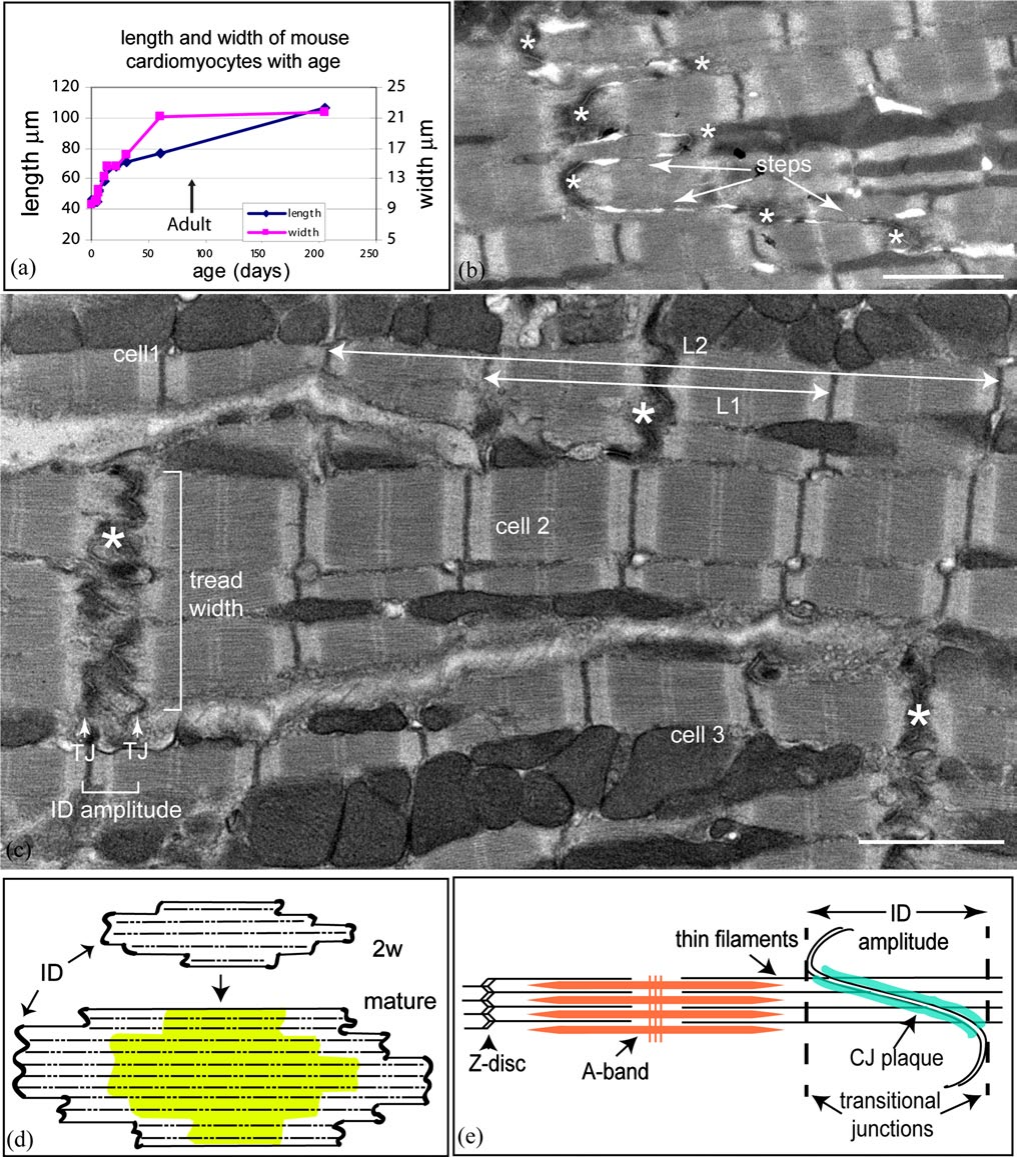
**a** Changes in mouse (strain C57Bl6) cardiomyocyte size with age (data taken from Leu et al. [26]; LV and RV data are averaged; thickness and depth data averaged for width of cells). **b** Longitudinal view of 2-week control (mouse strain sv129) papillary muscle showing narrow lateral width of ID treads (*asterisks*) between steps (*arrowed*), *Scale bar* 2 μm. **c** Electron micrograph of papillary muscle from a 6-month-old control heart showing the ID at the junction between three cells. The folded tread regions are indicated by *asterisks*. The lines *L1* and *L2* indicate the measurements made on micrographs to calculate the amplitude of the ID (see “Materials and methods”). TJ (*white arrows*) indicates the position of the transitional junctions (TrJ) between the proximal sarcomere and the folded ID. The *bracket* between them indicates the ID amplitude. The width of an ID tread is also shown by a bracket, *scale bar* 2 μm. **d** Diagram showing the proposed mechanism of cardiomyocyte growth. The 2 weeks cell is narrow with multiple ID steps at the two ends. As the cell matures, the cell widens (indicated by the *green area*) and elongates at the ID ends of the fibrils as shown by the *white areas*. The pattern of ID steps in the older cell is similar to that in the young cell. **e** Diagram showing the position of the TrJ at the margin between the myofibril and ID and the distance defined as the ID amplitude. Also shown is the relationship of the thin filaments of the final sarcomere extending into the CJ on the ID membrane folds

The heart grows by coordinated increases in the size of the chambers and the thickness of the walls to allow an increase in ejected volume. Most of this change is due to an increase in size of cardiomyocytes. However, little is known about the mechanism of their lateral or longitudinal growth. In mouse heart, in the first 4 days after birth there is a significant proliferation of myocytes followed by a hypertrophic phase with a concomitant change in both length and width of the cells [26]. This growth is rapid for the first 2 weeks. Subsequently, the width continues to increase until early adulthood (2–3 months) and then plateaus in the absence of growth pressures such as pregnancy or exercise [20]. In contrast, the lengthwise growth slows after 2 weeks but continues at a rate of about one sarcomere every week throughout life. The early growth phases up to 4 weeks were plotted by Leu et al. [26]. Figure 1a extends that plot to old age using other data from Leu et al. and clearly shows the decrease in the rate of growth after reaching maturity.

Cardiomyocytes are integrated with each other at their ends through their intercalated discs (ID), a complex region essential for electrical, mechanical, and other signaling communication between the cells and, hence, heart function and growth. The ID takes the form of a stepped structure [13]. The membrane at the transverse treads of the ID steps is deeply folded or plicate (asterisks in Fig. 1b, c). A notable feature of the 2-week-old cardiomyocytes is the small lateral width of the treads or plicate regions across the muscle cell between longitudinal steps (Fig. 1b). The cells themselves are narrow and the plicate regions are sometimes only an angled adherens junction (AJ) between cells. The mature muscle, in comparison, has more established treads of a greater breadth (Fig. 1c). These observations suggest that lateral growth of the cell is coordinated with lateral extension of the ID treads. In addition, it has been suggested that longitudinal extension occurs by sarcomere addition at the ID [4, 6, 54]. A diagram illustrating growth from 2 weeks to maturity incorporating these features is shown in Fig. 1d. To test this mechanism, we have further investigated the role of the ID in cardiomyocyte growth in mouse and man.

The major structural element of the ID membrane is the AJ, which lines the slopes of the folds with a protein-rich “plaque”. Into this are inserted the elongated thin filaments from the terminal sarcomeres (Fig. 1e) and, hence, the tension generated during contraction is transmitted across the membrane here [13, 53]. These junctions have been found to contain, in addition to AJ proteins (e.g., β-catenin, N-cadherin), some desmosomal proteins (e.g., desmoplakin, plakoglobin) and have been called areae compositae [7, 14] or hybrid AJ [28]. We will refer to them as composite junctions (CJ) here.

It is remarkable how well ordered the sarcomeres at the ends of the myofibrils are in spite of differences in amplitude of the ID folds. In agreement with this, we have identified, at the margin between the ends of the myofibrils and the beginning of the ID folds, a transitional junction (TrJ) comprising a myofibrillar and a membrane-associated region (Fig. 1c, e) [4, 6]. The membranous TrJ is associated with the peaks of the ID folds and the cytoskeletal protein αII spectrin. The myofibrillar TrJ is rich in selected Z-disc proteins, such as α-actinin, titin, and ZASP, but lacks the normal density of the Z-disc. It is suggested that the myofibrillar TrJ acts as a proto Z-disc and that a sarcomere could be inserted here when the amplitude of the membrane fold allows. Support for this mechanism comes from the work of Yoshida et al. [54] on the rabbit heart under pressure overload. They showed by electron microscopy that in a cycle of new sarcomere addition, there were concomitant changes in morphology of the ID membrane. In particular, at one stage, the ID folds increase in amplitude from ~0.6 to ~2 μm when new sarcomeres become incorporated. It would be expected that in a continuously growing heart, all the morphological states described by Yoshida and his colleagues should be seen.

The Yoshida intervention in rabbit heart accelerates growth and leads to a dilated cardiomyopathy (DCM), which is reversible. It is of value to compare their observations to clinical examples of DCM hearts. In DCM, there appears to be a breakdown in the coordinated growth of the heart; the ventricular walls thin and balloon such that the pressure the heart can exert is reduced [15, 44]. The condition results in or from myocyte disorder, myofibril disarray, and fibrosis, and can lead to heart failure and death. One common feature of these samples is that the ID fold amplitude is much greater in DCM than in healthy heart [35]. Other evidence that implicates the ID in DCM is that specific mutations of CJ proteins have been associated with DCM in human hearts [23]. Furthermore, mouse lines that suffer with DCM show changes in expression of CJ proteins compared to controls [10, 21, 27, 35, 45, 55].

To further understand the changes in the ID during aging and disease (DCM), we have used two of these mouse models, the MLP null, and one expressing a heart-specific β-catenin mutant (cΔex3). Muscle LIM protein (MLP) is a small signaling protein expressed in smooth and cardiac muscle cells that is thought to be a signal transducer in mechano-signaling [16, 51]. MLP-null mice that survive the critical 2 weeks after birth live to a normal mature age [1]. In the adult heart, the cells are more heterogeneous in size and shape than in the controls [26]. We have recently described the other mouse line, cΔex3, in which a mutant β-catenin is specifically expressed in the left ventricle (LV) of the heart after birth under the control of the myosin light chain 2v promotor [21]. The mutant protein has a deletion of exon 3, which in normal protein can be phosphorylated to control turnover. The cΔex3 have no pathology postnatally, but progressively develop DCM and die from the heart failure at about 4–5 months [43]. Of further interest here is that lateral growth is affected, the cross-sectional area of the cardiomyocytes in the cΔex3 mutant varies strongly with an average twice that of the control at 2 months.

There are many elegant descriptions of ID structure in the literature (see [13]). However, there is little or no quantitative measure of the amplitude or lateral width of the plicate regions. Here we describe a high-resolution electron microscopy investigation of samples of control and DCM hearts from both human and mouse for insights into the mechanism of lateral growth and sarcomere addition at the ID. We have carried out a quantitative analysis in mouse of ID fold amplitude distribution and lateral step width to monitor how these vary with age and heart compartment. We find further support for our model of growth (Fig. 1) and for the idea that cardiomyocytes elongate initially by increases in fold amplitude followed by sarcomere addition. We have also characterized structural changes that occur in high-amplitude IDs and find evidence in the mouse DCM models that the process of sarcomere addition appears to become stalled and that step formation and lateral growth of the treads at the ID is compromised.

## Materials and methods

### Tissue

#### Mouse tissue

MLP-null mice were as described by Arber et al. [1]. SV129 strain mice were used as controls. The cΔex3 mice, in which exon 3 of β-catenin is conditionally deleted in the LV of the heart were as described by Hirschy et al. [21]. These mice are raised on a mixed background of C57Bl6/SV129. Similar mice that were floxed, but were cre negative, were used as controls.

#### Human tissue

Human tissue was obtained from the Human Heart Tissue Bank, University of Sydney, Australia. Five samples of IDCM heart and five donors were used. The tissue was supplied frozen and stored in liquid nitrogen. This work was carried out under relevant ethical permission and fulfilled all requirements of the Human Tissue Act (UK).

### Electron microscopy

Specimens for electron microscopy were prepared as previously described [5]. Briefly, after the animals were killed, the heart was exposed and perfused with PBS containing 20 mM 2,4, butanedione monoxime (BDM) as a relaxant followed by 4 % PFA in PBS. After excision, the heart was cut transversely into 1-mm slices. Samples for microscopy were taken from the two central slices through the ventricles. For human heart material, 20-μm cryosections were collected on coverslips and washed in PBS. Human and mouse specimens were further fixed in glutaraldehyde followed by osmium, dehydrated in ethanol, and embedded in Araldite.

Sections of 70 nm were cut, where possible, with the fiber axis parallel to the knife edge to minimize section compression in this direction. Sections were stained in 2 % KMnO_4_ and lead citrate before viewing in a Hitachi 7600 transmission electron microscope equipped with an AMT HR/HRB digital camera and HMT software in the Centre for Ultrastructure Imaging, King’s College London. Images for ID amplitude measurements were taken at a nominal magnification of 10 K (14 nm/pixel).

### Immunofluorescence

Ultra-thin cryosections (200 nm) of sucrose-infiltrated formaldehyde-fixed tissue were cut and stored as previously described [48]. Sections were incubated in primary antibody in 1 % BSA for 1 h at room temperature before washing and incubating in secondary antibody for 30 m. Details of primary antibodies, αII spectrin, β-catenin, connexin 43, α-actinin, and secondary antibodies were as described previously [6]. Rabbit polyclonal anti-α-catenin (C-2081) was from Sigma-Aldrich, UK. Sections were viewed on a Zeiss Axiovert 200 light microscope.

### Western blots

Western blots were carried out as described [46]. Whole mouse hearts or small samples of human heart that were frozen were pulverized and immediately dissolved in sample buffer. Aliquots of approximately the same protein concentration were loaded onto 10 % SDS gels for electrophoresis. Gels were blotted onto nitrocellulose and probed for β-catenin and tropomyosin (TM) at the same time using rabbit polyclonal anti-β-catenin (C-2206, Sigma, UK) and mouse monoclonal anti-TM (T 2780, Sigma, UK). Digitized blots were analyzed using ImageJ software.

### Analysis

#### Amplitude of the ID

Measurements of the amplitude of the folds of the ID were obtained using the analysis software on the Hitachi 7600. We used our previous observation [6] that at the end of the terminal sarcomere, next to the ID, there is a zone, the TrJ, which is located where the Z-disc would be expected to be although there is no Z-disc density. We define the amplitude of the ID as the distance between the TrJs. We estimate this on well-ordered longitudinal regions where at least two sarcomeres to either side of the ID could be seen, by measuring the distance corresponding to two sarcomere lengths plus the ID (*L*_1_) and four sarcomere lengths plus the ID (*L*_2_) (see Fig. 1c). The ID amplitude is calculated as (2 × *L*_1_ − *L*_2_). In addition, the sarcomere length, (*L*_2_ − *L*_1_)/2, was determined. This gave an average value of 2 μm (SD ± 0.2 μm, *n* = 1,560) indicating that there was little shrinkage of the specimens due to fixation.

#### Fold separation

The ID membrane is folded into peaks so that it resembles an egg box. A transverse section through the tops of the folds shows a series of circular profiles (see for example, Fig. 4b). To estimate the distance between the peaks, the coordinates of the centers of the round profiles were obtained in ImageJ and the distance between the peaks calculated and analyzed in Excel. The distance to the nearest neighbor for each of the peaks was extracted from the data. The average of these values was taken as an estimate of the nearest neighbor spacing.

## Results

### Amplitude variations in the ID tread in control mouse hearts

**Fig. 2:**
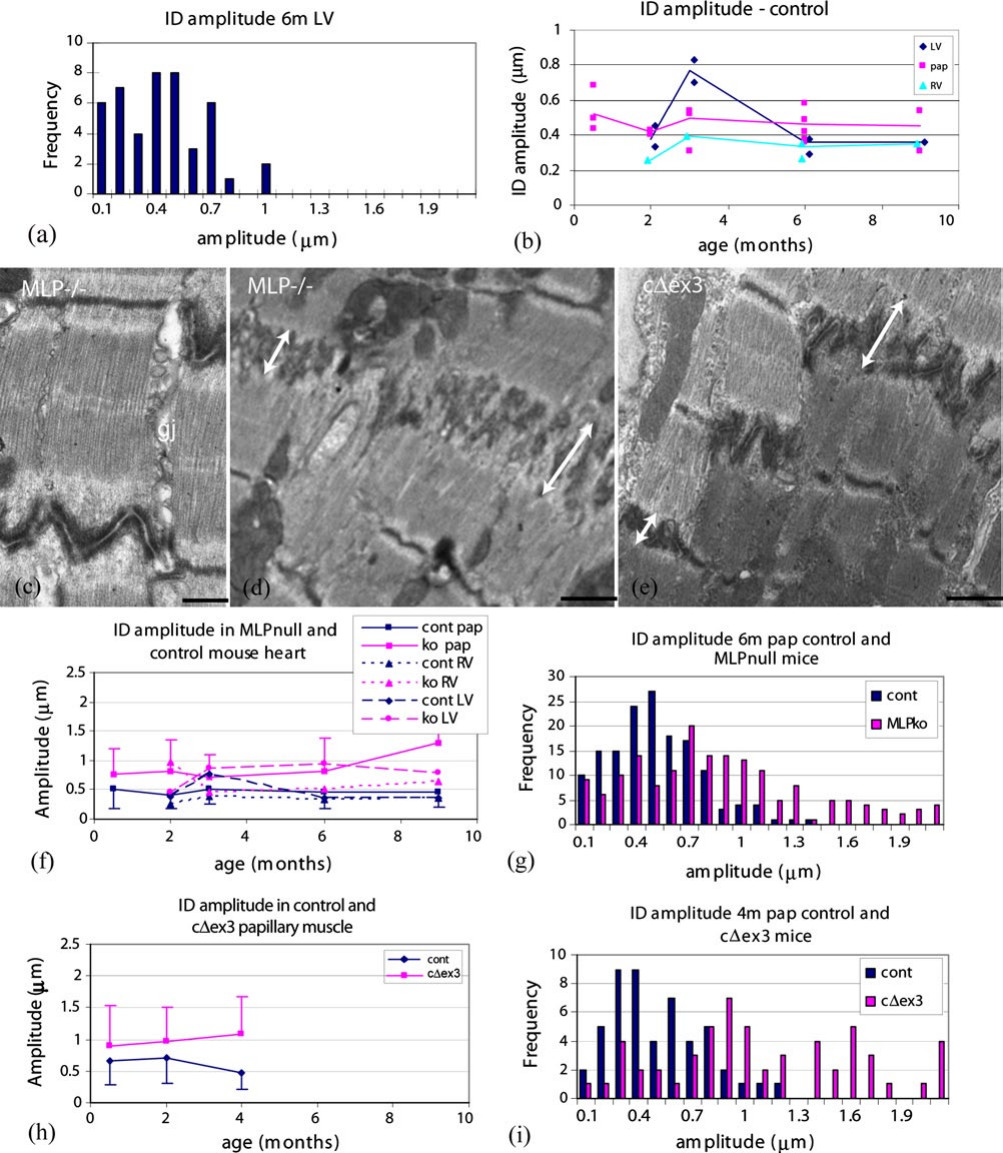
ID fold amplitudes. **a** Distribution of ID fold amplitudes in one electron microscope specimen of 6-month control (SV129) papillary muscle. **b** Plot of averages from areas such as shown in **a** for all control samples of all ages. *Lines* show averages of all data from any one heart compartment. *Diamond* LV, left ventricle, *triangle* RV, right ventricle, *square* pap, papillary muscle. **c**–**e** Electron micrographs of papillary muscles from mutant mouse lines showing the ID region. **c** 2-month MLP-null, *gj* gap junction. **d** 6-month MLP-null and **e** 4-month cΔex3 mutant. In **c** and **d**, *double-headed arrows* show variation in ID amplitude between two cells from one part of the ID to another, *scale bars* **c** 0.5 μm, **d**, **e** 1 μm, **f** plots of average ID amplitude versus age for all samples from different regions of heart from control and MLP null. *Error bars* show SD of data from papillary samples. **g** Distribution of ID amplitude in 6-month papillary muscle from control and MLP-null hearts. **h** Plots of average amplitude versus age for papillary muscles from control and cΔex3 mutant mice. *Error bars* show SD of data. **i** Distribution of ID amplitude in 4-month papillary muscle from control and cΔex3 mutant mice

Measurements of ID amplitude, i.e., the distance between the TrJs in neighboring cells, were made on longitudinal sections of mouse heart viewed in the electron microscope as described in “Materials and methods” (Fig. 1c, e). The distribution of values from a small area (~1 mm^2^) of 6-month LV muscle is shown in Fig. 2a. The average measurement is 0.38 μm (SD ± 0.24 μm, *n* = 45). The standard deviation of the measurements is typically about one-half of the average value. This relatively narrow distribution can be understood in terms of the close association of groups of cells and the relationship between Z-discs and the equivalent TrJs at the edge of the ID. At the junction between any two cells there may be several longitudinal steps of one or more sarcomere lengths (Fig. 1b, c). In this case, each plicate or folded region has essentially the same amplitude. In addition, any one cell contacts several cells at each end. The fibrils in neighboring cells are often aligned axially so that their Z-discs and hence their TrJs occur at the same axial levels. This dictates that the amplitude of the ID will also be similar in a locally overlapping group of cells. However, we find that the average amplitude in any one area can vary significantly from that in another part of the same heart or from one mouse to another.

Although there is local variation of the ID amplitude within a sample, it is not clear from the literature whether the range observed varies with age or the heart compartment. We have investigated this in RV, LV, and papillary muscle in immature hearts at 2 weeks at the end of the stage of rapid hypertrophy, as well as mature 2-, 3-, 4-, 6-, and 9-month-old animals (strain sv129). The data points plotted in Fig. 2b show the average amplitude obtained for each EM sample examined against the age of the animal for the three different compartments. For each age, there is a significant range of values. The* lines* show the average of all amplitudes measured for each type of specimen with age. Most samples were papillary muscle since here the cells are all oriented in the same direction and it is easier to obtain longitudinal views to analyze. Taking the average measurements from papillary muscle at each age group (Table 1), there is no significant difference between them even at 2 weeks (0.67 > *p* > 0.04 for all pair-wise age comparisons). Data from the fewer samples of LV and RV confirm that there is little evidence for a temporal variation in ID amplitude in the normal mature heart. Pooling the data for mature samples (ages 2–9 months) gives similar values for papillary and LV at 0.46 μm (SD ± 0.25 μm, *n* = 326 measurements from seven animals) and 0.44 μm (SD ± 0.29 μm, *n* = 243, four animals) respectively, while the RV average is somewhat smaller at 0.34 μm (SD ± 0.19 μm, *n* = 134, four animals).

**Table 1:** ID amplitudes in papillary muscles

	2 weeks	2 months	3 months	6 months	9 months
Control	0.52 ± 0.34; *n*_d_ = 82^a^; *n*_a_ = 4^b^	0.42 ± 0.23; *n*_d_ = 57; *n*_a_ = 1	0.49 ± 0.25; *n*_d_ = 57; *n*_a_ = 2	0.46 ± 0.27; *n*_d_ = 151; *n*_a_ = 3	0.45 ± 0.24; *n*_d_ = 62; *n*_a_ = 1
MLP null	0.77 ± 0.43; *n*_d_ = 96; *n*_a_ = 8	0.81 ± 0.55; *n*_d_ = 211; *n*_a_ = 5	0.72 ± 0.37; *n*_d_ = 86; *n*_a_ = 2	0.82 ± 0.54; *n*_d_ = 169; *n*_a_ = 4	1.30 ± 0.71; *n*_d_ = 41; *n*_a_ = 1
*P* ^c^	1.7E−05	6E−14	2.5E−5	7.4E−13	2.1E−9

	2 weeks	2 months	4 months		
Control	0.65 ± 0.36; *n*_d_ = 19; *n*_a_ = 2	0.71 ± 0.41; *n*_d_ = 77; *n*_a_ = 4	0.47 ± 0.26; *n*_d_ = 50; *n*_a_ = 6		
cΔex3	0.89 ± 0.64; *n*_d_ = 23; *n*_a_ = 2	0.97 ± 0.53; *n*_d_ = 70; *n*_a_ = 3	1.08 ± 0.60; *n*_d_ = 56; *n*_a_ = 6		
*P*	0.145	0.001	1.5E−9		

Data μm ± SD

^a^Number of measurements

^b^Number of specimens

^c^ *P*, result of two-sample *t* test assuming unequal variances of age-matched data from control and mutant samples

We conclude that there is a broad distribution of ID amplitudes at all ages in support of the idea that there is natural variation that would accommodate continuous growth of the heart. However, the values fall mostly in the range from 0.2 to 1 μm with few of the bigger sizes of ~2 μm seen by Yoshida et al. [54] in their rapidly growing pressurized rabbit hearts. Therefore, we have examined the IDs of greater amplitude found in mouse models of DCM [10, 35].

### Amplitude of ID folds in DCM mouse hearts

Although as previously reported there are areas of fibrosis and sarcomere disruption in the DCM hearts of the MLP-null and cΔex3 mutant mouse models [1, 21], there are large areas especially in young mice where the muscle order is well maintained. Here, there are regions of high amplitude IDs where comparison with the control may give clues to the mechanism of sarcomere insertion if not the cause of the breakdown of order.

#### Fold amplitude in MLP-null hearts

ID amplitude ranges between two extremes in the MLP-null heart (Fig. 2c, d). The stepped narrow ID, complete with a gap junction, of Fig. 2c is very like control muscle, whereas the very thick ID of Fig. 2d shows significant differences from the control. In particular, the amplitude within one ID between two cells can vary significantly (double-headed arrows).

As in the control heart, locally the measurements do not vary so much, although both the average value and the variation are usually greater than for the control. Figure 2f shows a plot of the average values at each age and for each compartment compared to control. As in the control, there is no significant difference between the MLP papillary averages at different ages, except possibly at 9 months where there are more disordered regions with big IDs that are difficult to measure. However, there is a significant difference between ID amplitudes in control and MLP null at all ages even at 2 weeks before the hearts show obvious signs of hypertrophy and dilation [10] (Table 1). The fewer data from the LV and RV in general supports these conclusions.

The range of ID amplitude measurements at all ages is much greater in the MLP null than in the control as can be seen from the error bars (standard deviation) in Fig. 2f. Histograms of all the data for 6-month papillary muscle from control and MLP null shows that the two ranges overlap from ~0.1 to 1 μm but many of the MLP measurements are greater (Fig. 2g). However, there appears to be an upper limit to the possible size of an ID; the range does not extend significantly higher than 2 μm.

#### Fold amplitude in cΔex3 hearts

Images of IDs in cΔex3 mature papillary muscle show similar features to MLP-null hearts (Fig. 2e). Again, the average ID amplitude is generally greater in the hearts expressing mutated protein than in the controls and the distribution of measurements is wider (Fig. 2h, i). Table 1 shows that average values in cΔex3 papillary muscle at 2 weeks are not significantly different than controls, as would be expected, since there is probably little expression of mutant protein at this age [21]. However, averages at 2 and 4 months are increasingly significantly greater than the controls (mixed breed sv129/C57BL6) (Fig. 2h). This corresponds to progressive increases in heart dilation in cΔex3 animals, while the MLP-null animals show the stabilization of heart function at this age [43]. Again, the histograms of combined data from 4-month control and cΔex3 animals show that the relatively broad distribution in control is broadened further in the mutant with many measurements near, but again not much greater than 2 μm (Fig. 2i).

### Sarcomere addition at the ID

**Fig. 3:**
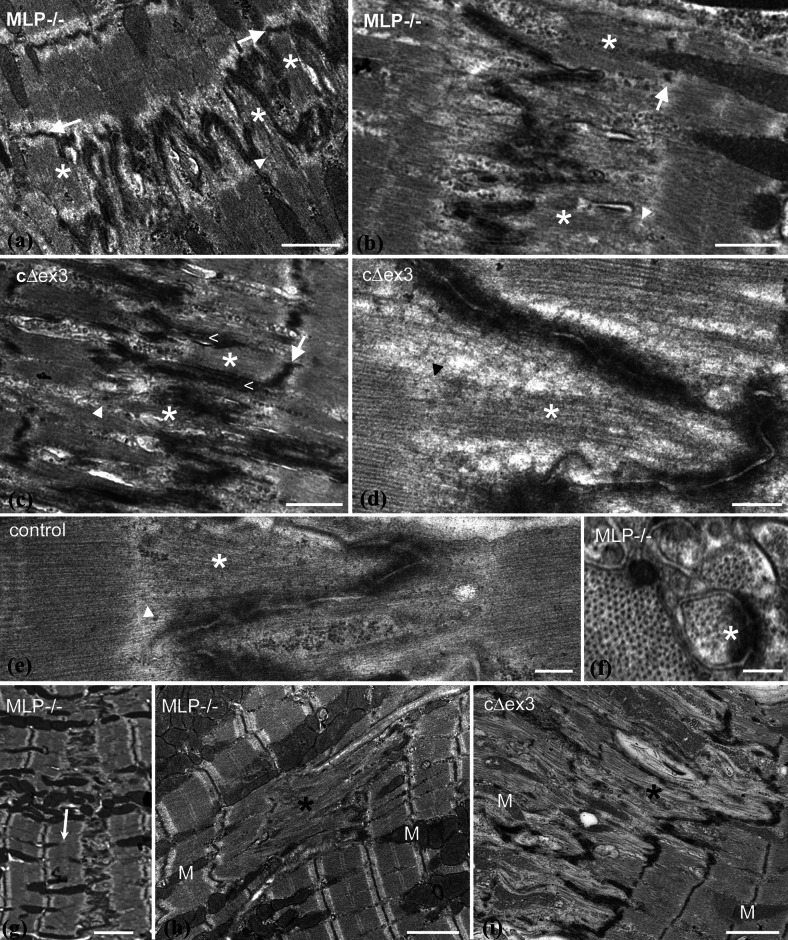
Longitudinal sections of high-amplitude IDs showing insertion of thick filaments or A-bands into grooves. **a** 6-month papillary MLP-null, **b** 2-month RV MLP-null, **c** 4-month papillary cΔex3, **d** 2-month papillary MLP-null, **e** 2-week papillary control; *asterisks* show A-bands or thick filaments in folds. *White arrows* Z-discs at the TrJ position. *Arrowheads* no Z-disc at TrJ. *Less than symbol* in **c** indicates membrane “spikes” at edge of new sarcomere. *Scale bars* **a**–**c** 1 μm, **d**, **e** 0.25 μm. **f** Transverse view through a peak of ID showing lattice of thin and thick filaments within a fold (*asterisk*) from 9-month MLP-null papillary muscle, *scale bar* 0.2 μm. **g** Low magnification longitudinal view of 6-month LV from MLP null showing one-sided step in ID amplitude and insertion of sarcomeres (*arrow*). Longitudinal views showing disruption of sarcomere order (*asterisks*) to one side of the ID in MLP-null 6-month papillary (**h**) and cΔex3 4-month papillary (**i**), *M* mitochondria, *scale bar* **g**–**i** 2 μm

Thin actin-containing filaments are normally the main feature within the folds of the ID. However, in the longer folds of amplitude 2 μm or so, extra density from the presence of thick filaments is also sometimes apparent (Fig. 3a–f, asterisks). Such regions are most often seen in DCM samples (Fig. 3a–d), but are occasionally found in control hearts (Fig. 3e). Usually, a fully fledged sarcomere within a fold with a defined A-band and a Z-disc (white arrows) at the level of the TrJ is seen (Fig. 3a–c). Sometimes, there are A-bands or groups of thick filaments in grooves where the Z-disc is less well developed or absent (Fig. 3a–e, arrowheads). The presence of the thick filaments within the folds is confirmed in transverse section by the appearance of a thick/thin filament lattice within a circle of an ID membrane fold (Fig. 3f).

In rabbit heart under pressure, Yoshida et al. [54] identified enlarged folds or grooves containing sarcomeres that alternated in abutting cells. We also observe that sarcomere-filled folds can alternate from one cell to the next (Fig. 3a). To either side of the new sarcomeres there are longitudinal “spikes” of membrane (Fig. 3c).

### Other observations that support growth at the ID in DCM hearts

In DCM hearts, but rarely in controls, the amplitude of the ID between two cells varies significantly as previously noted. Often, this results in the insertion of an extra layer of sarcomeres at a step in the ID in one cell only (Figs. 2d, 3g). The latter situation is seen more frequently in the MLP-null heart.

Further evidence that the ID is involved in DCM is found in cells where there is a loss of the sarcomere relationship at the ends of cells. It is clear from most of the micrographs presented here that despite variations in the ID, the organization of the sarcomeres including the last one before the ID is very well maintained; an order that points to the robustness of the TrJ. However, on occasion this changes. Figure 3h and i show regions of cells from MLP-null and cΔex3 mutant heart where the ordered arrangement is lost. These regions occur next to IDs that are always very wide. Interestingly, the cell to the other side of the ID is usually quite normal, indicating that the disordered growth has been one-sided. The areas of disorder shown are small but they can be very large. They are usually regions of supercontracted fibrils where t-tubule and mitochondria organization is disrupted. Indeed, in MLP-null hearts, mitochondria are missing from these areas. Clumping and absence of mitochondria has previously been reported in MLP-null hearts as has loss of function [49, 52].

### Tread widths and lateral growth

It was noted above that the plicate tread regions of the ID between longitudinal steps in 2-week hearts are narrow compared to the mature muscle (Fig. 1b, cf. Fig. 1c). In some cases, especially in DCM hearts, the mature IDs can reach across a cell with few steps (e.g., Figs. 2c, 3g). This suggested that when the cross-sectional area increases as the myocyte matures, the plicate regions are laterally widened. To investigate this relationship, we have measured the lateral extent of these regions in longitudinal sections of 2-week, 2- and 6-month papillary muscles in control and MLP-null and 2-week, 2- and 4-month muscles in control and cΔex3 hearts. The data in Table 2 show that for the control animals, there is an increase in the average step width from 2 weeks to 2 months and little or no change after this. This correlates with the observed twofold increase in cross-sectional area of the cells between 2 weeks and 2 months but little increase later, and indicates that the number of steps at the ID remains relatively constant during both maturation and subsequent aging. For the mutant specimens, again, there is an increase in tread width between the 2-week and 2-month age group, but unlike controls, there is a further significant increase with age with a much wider distribution, indicating a loss of steps in the older heart.

**Table 2:** Lateral width of plicate regions between steps at the ID

	Control MLP	MLP null	Control cΔex3	cΔex3
2 weeks	1.52 ± 0.74 *n* = 172	1.50 ± 0.84 *n* = 172	1.92 ± 1.29 *n* = 56	1.58 ± 0.73 *n* = 46
2 months	2.15 ± 1.22 *n* = 32	2.80 ± 1.45 *n* = 80	2.30 ± 1.48 *n* = 129	2.12 ± 1.35 *n* = 112
4 months			1.82 ± 1.05 *n* = 112	3.10 ± 2.24 *n* = 173
6 months	2.52 ± 1.23 *n* = 110	4.01 ± 2.11 *n* = 75		

Data μm ± SD

### Structural changes at the ID membrane with amplitude

**Fig. 4:**
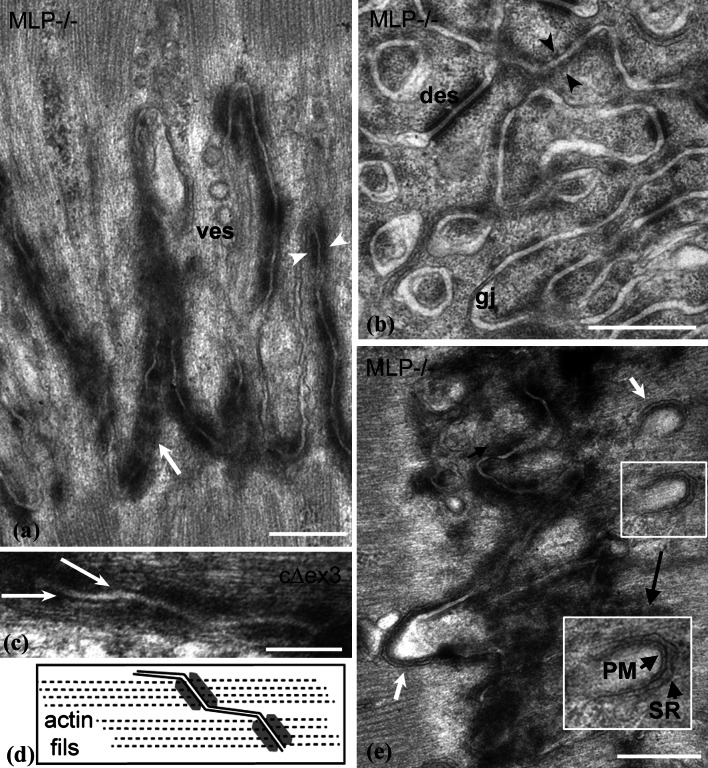
**a** Longitudinal view of high-amplitude ID membrane folds showing the breakup of the dark plaque into punctate regions (*white arrow* and *arrowheads*) with clear stretches of membrane between in 6-month MLP-null LV. Many coated vesicles are seen (*ves*). **b** Transverse view through ID folds in 9-month MLP-null RV illustrating circular profiles and breakup of the CJ plaque. *Arrowheads* show symmetrical distribution of plaque spots across the membrane; *des* desmosome, *gj* gap junction. **c** Small area of ID membrane from 4-month cΔex3 papillary showing thin filaments running into punctate plaque on angled regions of membrane. The clear membrane regions are more or less parallel to the thin filaments. **d** Diagram illustrating the path of the thin filaments in **c** and the change of angle of the membrane. **e** View of exaggerated loops at the top of ID folds seen in 3-month LV from MLP null (*white arrows*). *Insert* at higher (1.5×) magnification shows close association of an SR vesicle (*SR*) with plasma membrane (*PM*), *scale bars* **a**, **b**, **d** 0.5 μm, **c** 0.2 μm

The ID membrane of MLP mice and other DCM specimens is more convoluted than in the wild type [10] but does the fold frequency increase? We have estimated this by measuring the separation, in transverse views such as Fig. 4b, of the circular sections through the folds (see “Materials and methods”). The average nearest neighbor separation was calculated for 3- and 9-month control and MLP-null papillary and LV. The values are essentially the same for all specimens analyzed at ~0.5 μm (SD ± 0.2 μm), approximately equivalent to the diameter of the “fibrils” in cardiac muscle. Similar values were obtained for 4-month cΔex3 papillary muscle and their controls. Hence, although the folds are amplified in DCM, they do not become laterally wider.

At higher magnifications, other features of the ID membrane become apparent. In IDs with small amplitude, the CJ plaque is more or less uniformly dense and covers the slopes of the folds (Figs. 1c, 2c). In larger amplitude IDs from all specimens, the plaque appears more broken up. The punctate nature of this distribution is revealed in both longitudinal and transverse sections (Fig. 4a, b). The plaque clumps are in close proximity in adjacent cells so that the tension can be transmitted directly across the membrane to the neighboring cell through thin filaments (arrowheads Fig. 4a, b). The plasma membrane between these “spot welds” is uncoated and the membranes of neighboring cells have a greater separation. In very wide IDs, these stretches of membrane are more parallel to the longitudinal axis than the punctate plaque regions. The latter are clearly angled to the thin filaments (Fig. 4c, d).

Also associated with these high-amplitude IDs are more extended lengths of uncoated membrane. Here there are numerous vesicles, many of them coated (Fig. 4a).

### The transitional junction in wide IDs

The TrJ membrane compartment at the tops of the folds was shown previously to be clear of plaque [6]. In the thick IDs of DCM hearts, the tops of the folds are still clear of plaque but sometimes they are exaggerated into relatively empty loops of plasma membrane (Fig. 4e arrowed). These loops are often closely associated with peripheral couplings of the junctional sarcoplasmic reticulum (SR) (insert Fig. 4e) [9, 12].

**Fig. 5:**
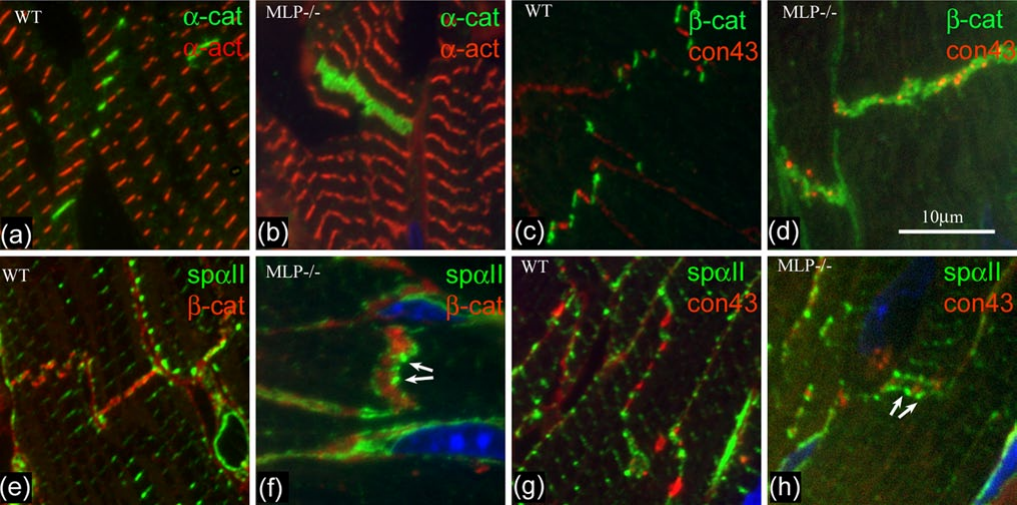
Immunofluorescence of sections of control (**a**, **c**, **e**, **g**) and MLP-null (**b**, **d**, **f**, **h**) papillary muscle labeled with various antibodies. **a**, **b** α-catenin (*green*) and α-actinin (*red*). **c**, **d** β-catenin (*green*) and connexin 43 (*red*). **e**, **f** αII spectrin (*green*) and β-catenin (*red*). **g**, **h** αII spectrin (*green*) and connexin 43 (*red*). *White arrows* in **f** and **h** indicate loops of spectrin label

To see whether the TrJ proteins are maintained in the bigger IDs in DCM heart, we have used immunofluorescence to investigate the distribution of α-actinin and αII spectrin, proteins characteristic of myofibrillar and membranous TrJ, in relation to the CJ and gap junction proteins α- or β-catenin and connexin 43 (Fig. 5). Labeling for the CJ proteins, α-catenin, or β-catenin, shows thickening of the ID in MLP null compared to control (Fig. 5a, b, e, f). α-Actinin is split across the ID in Fig. 5a and b but forms a wider doublet in the MLP-null example. Similarly, αII spectrin label reveals a much wider doublet in the MLP null (Fig. 5f, h) compared to controls (Fig. 5e, g) [6]. Furthermore, it is not a continuous line, rather, the punctate spots (arrows Fig. 5f, h) resemble the distribution of the inflated loops of ID membrane coated with SR described above (Fig. 4e).

Figure 5c, d, g, and h show the distribution of connexin 43, a gap junction protein, with respect to β-catenin (Fig. 5c, d) and spectrin (Fig. 5g, h). In control mouse hearts, connexin is found on the longitudinal stretches of membrane at the ID steps, and is clearly separate from β-catenin on the membrane folds of the ID treads (Fig. 5c, cf. Fig. 2c). However, in the MLP heart, the connexin 43-associated structures are smaller and usually sandwiched in the β-catenin-rich regions. The same relationship is true for αII spectrin and connexin 43. There is little or no association of the two in the control whereas in the MLP null, the connexin is trapped in the tread regions of the ID. Small trapped gap junctions can be seen by electron microscopy (see Fig. 4b), which is consistent with previous observations that there is reduced connexin 43 in MLP heart [10].

### The ID in human heart

**Fig. 6:**
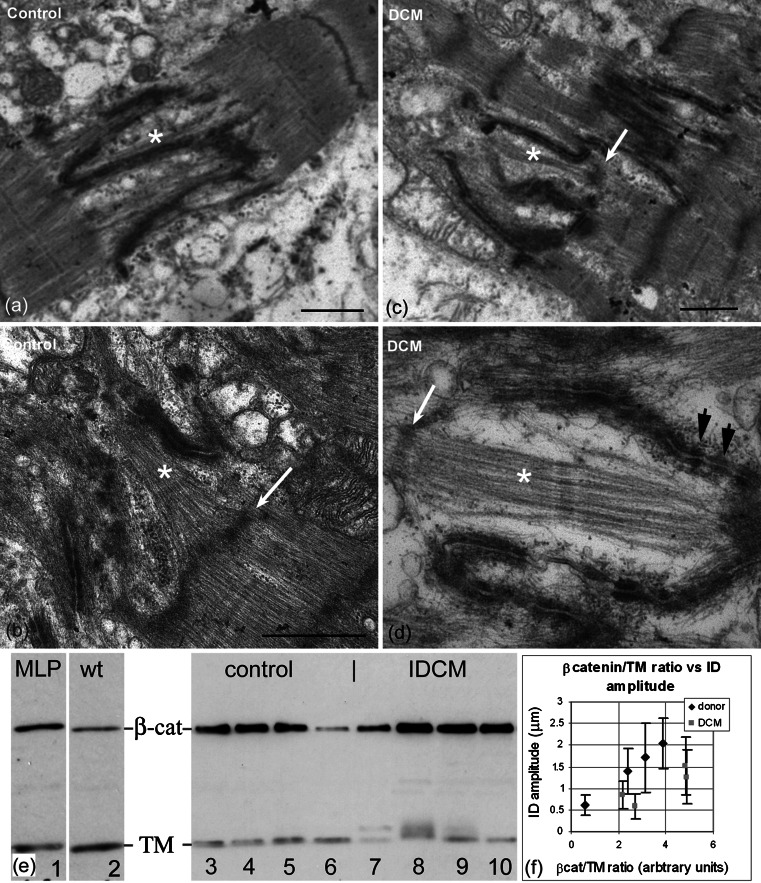
Electron micrographs of human left ventricle showing large amplitude IDs with evidence for sarcomere insertion. **a**, **b** control heart. **c**, **d** IDCM heart. *White asterisks* show A-band or thick filaments in grooves in the ID. *White arrows* show associated Z-discs at the TrJ level while there is no Z-disc evident in **a**. *Black arrows* in **d** indicate regions of punctate plaque on the membrane. *Scale bars* **a**, **b**, **d** 1 μm, **c** 0.4 μm. **e** Western blots of hearts labeled for sarcomeric tropomyosin (*TM*) and β-catenin (*β-cat*). *Lanes 1–2* mouse, *1* MLP null and *2* control. *Lanes 3–10* human LV, *3–6* control, and *7–10* IDCM. **f** Plot of ratio of β-catenin/TM compared to ID average amplitude measurements of LV samples of human heart, four control, and four IDCM. *Error bars* show SD in ID amplitude measurements

The observations above on mouse heart suggest that new sarcomeres are inserted at the ID when the membrane folds are sufficiently big (~2 μm). To establish whether this is the case in human heart, we have examined a number of control and IDCM LV samples. Unfortunately, because of ice damage, due to storing the tissue frozen and other factors, there is often structural disruption seen at the level of electron microscopy. However, well-preserved areas can be found. Here, significant variation in ID amplitude can be seen in both IDCM and normal tissue. Other features of the large amplitude IDs in the mouse, such as the break-up of the plaque along the ID membrane, are also seen (Fig. 6a–d). In some large-amplitude IDs (~2 μm) we see widened folds or grooves containing A-bands or bundles of thick filaments (asterisks, Fig. 6). Usually, there is a defined Z-disc at the TrJ position (white arrows, Fig. 6b–d). Occasionally, the Z-disc is not well developed or is absent (Fig. 6a). Therefore, the human tissue also shows evidence for sarcomere addition at the ID.

There is evidence that there are variable amounts of CJ proteins in DCM compared to normal hearts. In particular, in the mature MLP-null heart, the level of CJ proteins are doubled [10], whereas in the cΔex3 mouse heart there is little increase in CJ proteins except for the mutant β-catenin [21]. We have investigated this relationship in human heart samples by estimating the amount of CJ protein, β-catenin, in relation to the sarcomeric protein, TM, using Western blots (Fig. 6e). In agreement with Ehler et al. [10], control blots of MLP-null and wt mouse hearts confirm that β-catenin concentration is higher in the null (Fig. 6e, lanes 1, 2). In contrast, in human samples we see a considerable range of β-catenin concentration compared to TM in both IDCM and control hearts (Fig. 6e, lanes 3–10). However, in three of the four human IDCM hearts, the TM band was split, an effect not seen in the MLP-null mouse. Since the second band runs at a higher molecular weight than the normal, it is unlikely to be a degradation product. It is possible that it is the isoform of α-TM, TPM1-k, that has been recently identified in human hearts and that is upregulated in DCM [8, 38]. In chickens, this isoform is found in embryonic chick heart but not adult.

The range of β-catenin/TM ratios determined for control human specimens, though smaller on average, overlapped with that for DCM hearts. On comparing these values to measurements of the ID amplitude of the same specimens, we found that for each of the two types of sample, the β-catenin/TM ratio varied with ID thickness (Fig. 6b). However, the DCM samples have an increased ratio compared to the donors for the same ID amplitude. This supports the suggestion that there may be extra β-catenin associated with the DCM phenotype [35].

## Discussion

### Role of the ID in cardiomyocyte growth

We have investigated a model for cardiomyocyte hypertrophic growth from 2 weeks to maturity in which the ID is involved in increases in both cell width and length (Fig. 1d) and have found evidence to support this hypothesis. At 2 weeks postnatally, mouse cardiomyocytes are cylindrical, and the cell–cell connections have the intrinsic structure of the mature ID. As they mature they maintain axial ID connections at stepped intervals with several other cells with which they partially overlap laterally (Fig. 1d). This puts limitations on lateral growth since, for example, cells cannot all grow at their edges; this suggests, rather, general internal growth, which may result in widening and subsequent longitudinal splitting of fibrils. We have shown that lateral growth during maturation of the cells is not only accompanied by, but is correlated with, lateral growth of the ID treads so that the number of steps in the ID is approximately maintained.

**Fig. 7:**
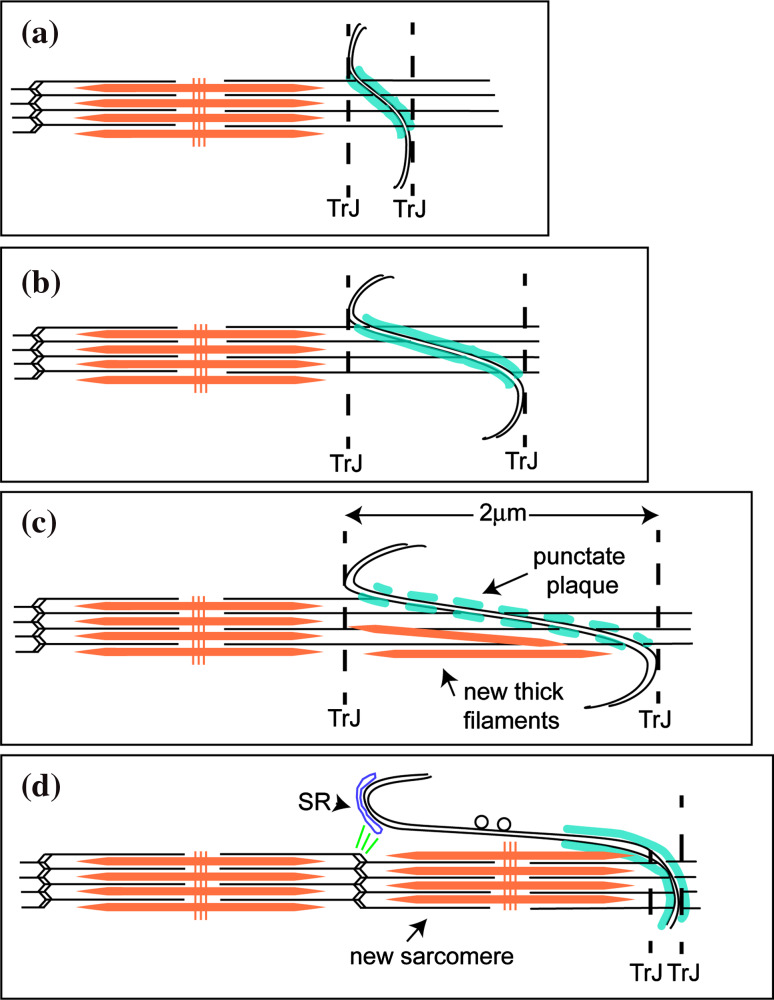
Proposed model of cardiomyocyte longitudinal growth. **a** ID with small amplitude. **b** ID folds increase in amplitude to allow cell growth and CJ plaque (shown in *turquoise*) becomes punctate (**c**). **c** At 2 μm, thick filaments and **d** whole sarcomeres are incorporated within a membrane fold. Vesicles are found on long stretches of uncoated membrane. The new Z-disc is associated with the SR at the top of the fold that may bud off to become a t-tubule. A new TrJ is established at the end of the new sarcomere

While the mature mouse cardiomyocyte width stabilizes at 2–3 months, the length steadily increases from 2 weeks into old age [26]. Our observations suggest that this growth occurs at the ID and the proposed mechanism is shown in Fig. 7 [4, 6, 54]. We find that in all types of specimens there is a naturally occurring wide range of ID amplitudes in all compartments of the heart, independent of age. In both young and mature control mice, the range is essentially from 0.2 to >1 μm, while in the DCM samples, the range increases to 2 μm. Our findings support the idea that changes of amplitude at the ID allow gradual elongation of the cells during heart growth. However, this method of growth is limited (Fig. 7a–c). We have never found IDs of amplitude significantly greater than ~2 μm, that is, one sarcomere length, indicating another means of growth at this stage. The micrographs suggest a mechanism of sarcomere addition within large amplitude folds.

It is possible that sarcomere addition occurs elsewhere in the myocyte but we and others have found little evidence for this. Growth at the end of the cell is supported by the presence in DCM mouse hearts of partial layers of sarcomeres or a proliferation of disordered sarcomeres at the ID. In addition, in vitro studies of sarcomerogenesis in neonatal cardiomyocytes suggest that fibrils develop further mature sarcomeres towards their ends [40]; a protofibril forms sarcomeres at its center that are flanked by mini sarcomeres that then mature.

In the mouse heart, at the beginning of the hypertrophic stage at 4 days, the oblate myocytes of the heart are obliquely associated through an elongated region of β-catenin-rich connections [22]. As the cardiomyocytes mature, these connections move to the ends of the cells to form the ID. This movement is a natural consequence of the model proposed here since the addition of sarcomeres to the ends of the fibrils would maintain the ID steps at each end of the cell as groups; the two separating as the cell grows as shown in the model in Fig. 1d.

Long ID folds in the control mouse heart where the growth is slow (~1 sarcomere/myocyte/week [26]) are rare. A possible reason is that extreme membrane extension and sarcomere insertion may normally be a short-lived process that is difficult to capture. At large fold amplitudes, the CJ plaque becomes punctate, and small, apparently unsupported, longitudinal regions of membrane result. These may be less stable and the tangential force on these areas may lead to more rapid incorporation of lipid and subsequent sarcomere addition as evidenced by large numbers of vesicles in the region.

### Possible mechanism of sarcomere insertion

Yoshida et al. [54] identified five phases of structural change at the ID in rabbit heart leading to the insertion of two sarcomeres, one on either side of the ID. We have not reliably seen these five phases nor do we have evidence for the addition of more than one sarcomere at a time but our observations support at least part of this mechanism. New sarcomeres are only seen in widened folds of the ID once they are >2 μm long in either of the abutting cells and spikes of excess membrane are seen to the side of the new sarcomere. Since all local cells have ID amplitudes of the same size, this would allow the coordinated insertion of a sarcomere across the whole interface of one cell with another, and even propagation across a whole group or syncytium.

We can consider the mechanism of sarcomere insertion in a little more detail (Fig. 7). The TrJ at the myofibril/ID border contains Z-disc proteins, α-actinin, N-terminal titin, and ZASP [4, 6]. Another protein with the same distribution about the ID is the nebulin-like protein N-RAP [10, 29]. These proteins appear to form a strong Z-disc-equivalent structure maintaining the myofibril order at the ID edge in all the specimens studied, independent of ID fold amplitude. At large amplitudes the long thin filaments running from myofibril into ID fold together with the proto Z-disc of the TrJ would appear to offer a suitable scaffold for sarcomere addition.

In their in vitro observations of embryonic and neonatal cardiomyocytes, Sanger and colleagues [40] describe a short sarcomere-like structure in the nascent myofibril that develops into a mature structure. At the beginning of this process, there are Z-bodies and thin filament bundles that may have some similarity to the TrJ and the actin array in the ID folds. However, in the heart, we do not observe short repeating structures in the ID folds or short thick filaments in small amplitude folds. Rather, in high-amplitude folds, which do not have Z-disc density at the TrJ, A-bands or groups of thick filaments are sometimes found. More often, in widened folds or grooves, sarcomeres are seen with complete A-bands and with Z-discs where the TrJ was. This suggests to us that thick filaments can be incorporated into the ID folds before the Z-disc is mature (model Fig. 7). It would be possible for filaments to be made in situ, slid in along the ID thin filaments, or brought to the ID by a microtubule-related process previously described [37]. The TrJ could act as an anchor to the new filaments by incorporating titin before maturing into a Z-disc with properly oriented thin filaments (Fig. 7c). Z-disc maturation after sarcomere formation has been observed in quail myotubes where, for example, incorporation of the titin binding protein, telethonin, is one of the latest processes to occur [50]. We have previously reported that neither of the Z-disc proteins, telethonin or its associate, FATZ, is present at the TrJ [4].

### Role of spectrin/SR/t-tubules in sarcomerogenesis

In addition to its myofibrillar, proto Z-disc, component, the TrJ also comprises the adjacent region including the tops of the ID membrane folds. These are bare of plaque and αII spectrin is found there [6]. Several other components of the spectrin-associated complex have been found at the ID such as βIIΣ2 spectrin [19], protein 4.1R [47], and ankyrin G [32]. Although none of these has been located precisely to the TrJ folds, protein 4.1R, like αII spectrin, does not overlap the distribution of β-catenin or connexin 43 at the ID [36]. The spectrin-associated membrane cytoskeleton acts as a scaffold for membrane-associated and trans-membrane signaling proteins (see [2]). Furthermore, several proteins that bind to the spectrin complex, such as MLP and filamins, have been found to interact with Z-disc/TrJ proteins [11, 31]. Hence, the spectrin scaffold is ideally placed to couple the fibrillar TrJ to the membrane TrJ at the ID.

In the MLP-null mouse, the tops of the ID folds enlarge into spectrin-rich loops often associated with junctional SR vesicles. It is possible that these loops of plasma membrane bud off and become t-tubules/SR at the Z-disc after sarcomere insertion. Certainly, αII spectrin has been identified in or near t-tubules [5, 25]. There is now evidence that t-tubules in cardiomyocytes from diseased hearts are disordered and incomplete, indicating that their association with the Z-disc is compromised [30]. The loss of t-tubules has been ascribed to changes in the Z-disc stress-sensing mechanism. MLP is one of the proteins implicated. It binds to spectrin, and other proteins of the Z-disc and the ID (TrJ) [10, 17]. A defect in the attachment of the ID membrane to the incipient new Z-disc at the TrJ in the mutant mouse heart could lead to a block in the mechanism of sarcomere addition and lack of propagation of forces laterally across the cell.

### DCM phenotypes

The DCM phenotype can be brought about by the abnormal elongation of cardiomyocytes [18]. However, others point to more variability in cell size in disease [3, 42]. The enforced DCM phenotype of the rabbit heart under pressure shows accelerated lengthening of the cells [54]. However, cardiomyocytes of hypertensive rats increase in both length and cross-sectional area with age [33, 41]. Certainly, the MLP-null and the cΔex3 mutant cardiomyocytes studied here do not grow significantly longer than cells in control animals, although the cΔex3 cells have twice the cross-sectional area at 2 months [21, 26]. However, both types show higher amplitude IDs that are rich in evidence of sarcomere insertion, suggesting that these hearts may reach a hiatus in one of the processes involved in sarcomere addition. That process may not be the same in the two models since morphological detail seen in the two are different (Table 3).

**Table 3:** Comparison between MLP-null and cΔex3 hearts

	MLP-null hearts	cΔex3 hearts
Similarities		
Cell size	More variable compared to control	More variable compared to control
	Same size on average as control	Same length on average cross-sectional area 2× control at 2 months
ID amplitude	Greater than control	Greater than control
ID fold repeat	Same as control	Same as control
NRAP	Increase	Increase
Differences		
Cell morphology	Brush-like at ends leading to loss of orientation of fibrils	Normal square morphology
ID tread and step morphology	Loss of steps with age. Big range of tread width	Loss of steps with age? Big range of tread width
Mitochondria distribution	More clumped, loss of ordered size, absence near ID	Approximately normal distribution
CJ proteins	Increase in CJ proteins β-catenin, cadherin, α-catenin decrease in connexin 43	Increase in mutated protein. Residual normal protein. Other CJ proteins normal
Fatality	Survival after 2 weeks postnatal to normal life span	Die at 4 months

Another feature in our DCM mouse hearts is the change in the number of steps at the ID and hence tread width, with age. Like controls, the maturing MLP null maintains a constant number of steps and achieves a modest increase in size while the cΔex3 mutant would need to increase the number of steps of the size measured to accommodate the large change of cell width observed. However, as the DCM model hearts age, the range and the average tread size increases significantly, with widths up to 20 μm observed in the cΔex3 heart. This implies a loss of ID steps. Our immunofluorescence and electron microscopy observations suggest that these steps, as evidenced by the presence of connexin 43 and gap junctions in control muscles, are absorbed into the high-amplitude ID treads in the MLP heart. Whatever the mechanism involved, broad IDs introduce a region of lateral stiffness different from that of the sarcomeric regions, which may have functional repercussions compared to spreading the ID segments axially.

A number of features described distinguish the MLP-null hearts from the controls and point to a lack of regulation in sarcomere addition and more dispersion in cell size (Table 3). These features point to a loss of communication at the ID both laterally within a cell and axially between cells. The latter is starkly revealed by the proliferation of disordered sarcomeres in one cell while its neighbor maintains its organized structure (Fig. 3h). These defects in sarcomere organization at the ID must result in a loss of strictly longitudinal tension transduction from cell to cell and divergence of myofibrils. This could explain the branching out at their ends and their bushy phenotype as previously suggested [10].

The cΔex3 mouse shows less gross structural disorder at the ID than the MLP null. The observation that the β-catenin-deficient conditional ko mouse grows and lives to a normal old age suggests that the mutant β-catenin is in fact the lethal element in the cΔex3 mice [21]. The excision of exon 3 leads to a loss of the phosphorylation site that controls wnt signaling and recycling or autophagic elimination of the normal β-catenin. In older animals, there is an overwhelming amount of mutant β-catenin (3 × normal at 2 months [21]) which accumulates at the CJs, presumably clogging the structure, preventing morphological reorganization, and inhibiting normal longitudinal growth, but apparently allowing unusual lateral growth and ID formation. Furthermore, the exon 3 deletion (res 5–80) may remove a major portion of the vinculin binding site found in the N-terminal 131 residues of β-catenin [34]. Consequently, the interaction of β-catenin with vinculin may be weak or absent, leading to modified interactions with actin at the CJ.

The loss of flexibility leads to early death in the cΔex3 mouse in contrast to the MLP null, where, in spite of the DCM phenotype, the cells are able to withstand the unusual pattern of forces that is inflicted on them and the animal can live to a normal old age.

### Role of β-catenin/CJ proteins at the ID

Mutations and changes of expression in ID proteins, especially those of the CJ, are associated with DCM [10, 21, 27, 28, 35, 45, 55]. In our mouse models, one or more of the ID junctional proteins is over-expressed. In other examples of DCM, there is a loss of such proteins. In the heart-specific N-cadherin knock out mouse, for example, there is a progressive loss of IDs with lethal results [24]. All of this evidence suggests that the absolute amount of adherens proteins is important and that within a range the ID is viable and the animal survives. However, too much or too little is fatal.

As in the MLP mouse, we found that in our human heart samples the average of the β-catenin/TM ratio in normal LVs was less than that found in the DCM samples. These observations might suggest that for normal hearts a standard amount of CJ proteins would be necessary to tether any one thin filament from the terminal sarcomere to the ID membrane. It is certainly clear that as the ID amplitude increases the plaque breaks up, and the CJ protein present in the punctate regions is adequate for the purpose. However, there was a significant range in the β-catenin/TM ratio in the human samples, indicating that a natural variation can be accommodated. An important consideration here is the form these punctate regions take. The precise composition of CJs and relationship between desmosomal and AJ proteins is not known. Some of these proteins can substitute for one another, e.g., plakoglobin for β-catenin [56] and the absence of some desomosomal proteins apparently leads to loss of adhesion of ID membrane leaflets [39]. The exact composition of the plaque therefore may significantly affect the strength of the membrane to membrane junctions and the attachment to actin and intermediate filaments; affects that could alter the process of fold extension and sarcomere addition.

## Conclusions

We have presented evidence for the insertion of sarcomere together with the SR/t-tubule at broad IDs in the heart. The evidence is compelling but still needs proof. While we have some ideas of how this could occur, there are many pressing questions such as what dictates that the ID folds have grown sufficiently, and that thick filament proteins should be made and incorporated into an A-band, how the Z-disc is matured, the SR/t-tubule formed, and how the excess ID membrane is removed and recycled.
